# Combining humanized mice, neurochemical, imaging, and cognitive biomarkers for drug discovery in mice.

**DOI:** 10.1002/alz70859_098668

**Published:** 2025-12-25

**Authors:** Anoosha Attaran

**Affiliations:** ^1^ The University of Western Ontario, London, ON Canada

## Abstract

**Background:**

The global population of senior citizens is growing rapidly, contributing to a rising prevalence of neurodegenerative diseases, including synucleinopathies such as Parkinson’s disease (PD), Lewy Body Dementia (LBD), and Alzheimer’s disease (AD). Drug development for synucleinopathies has been particularly inefficient. Progress in developing therapies targeting underlying mechanisms has been hindered by the poor translatability of animal models, which often fail to accurately reflect human synucleinopathy pathology, and the limited availability of cognitive biomarkers for predicting treatment outcomes. Our work aims to develop a comprehensive preclinical drug discovery pipeline that integrates humanized mice, neurochemical, imaging, and cognitive biomarkers to enhance predictive accuracy. This approach combines advanced behavioral assessments, imaging modalities, and real‐time neurochemical monitoring in behaving mice to better understand disease pathogenesis, identify therapeutic efficacy, and detect adverse effects.

**Method:**

We combined preformed fibril (PFF) injections of wild‐type human synuclein into mouse models expressing human synuclein with high‐throughput touchscreen‐based cognitive tasks relevant to synucleinopathies to investigate cognitive deficits. In parallel, fibre photometry recordings were used to measure dopamine dynamics and calcium signaling in freely behaving mice. Pathological changes were assessed using immunofluorescence, lightsheet microscopy, and MRI. We also initiated experiments to test the ability of different treatments, including drugs and genetic manipulations targeting chaperones, and a vaccine to mitigate alpha‐synuclein (a‐Syn) toxicity in these models.

**Result:**

Mice unilaterally injected with PFFs in the striatum exhibited significant cognitive deficits in touchscreen‐based tasks including pairwise visual discrimination (PVD) and visuomotor conditional learning (VMCL), which appear before motor impairments. Fibre photometry recordings revealed altered dopamine dynamics in freely behaving PFF‐injected mice. These cognitive and neurochemical changes were associated with elevated phosphorylated a‐Syn levels that spreads through cortical‐striatal‐thalamic networks, confirmed through immunofluorescence, Western blotting, and lightsheet microscopy. MRI analysis showed spatial atrophy patterns in PFF‐injected mice that mimic human synucleinopathy atrophy. Preliminary results indicate that treatments mitigate a‐Syn toxicity and improve cognitive deficits.

**Conclusion:**

We aim to establish a robust cost‐effective pipeline that improves the translation of preclinical discoveries to clinical success, ultimately accelerating the development of effective treatments for synucleinopathies and reducing the risk of late‐stage drug failures.

